# Improved PCR-Based Detection of Soil Transmitted Helminth Infections Using a Next-Generation Sequencing Approach to Assay Design

**DOI:** 10.1371/journal.pntd.0004578

**Published:** 2016-03-30

**Authors:** Nils Pilotte, Marina Papaiakovou, Jessica R. Grant, Lou Ann Bierwert, Stacey Llewellyn, James S. McCarthy, Steven A. Williams

**Affiliations:** 1 Department of Biological Sciences, Smith College, Northampton, Massachusetts, United States of America; 2 Molecular and Cellular Biology Program, University of Massachusetts Amherst, Amherst, Massachusetts, United States of America; 3 QIMR Berghofer Medical Research Institute, Brisbane, Australia; Johns Hopkins Bloomberg School of Public Health, UNITED STATES

## Abstract

**Background:**

The soil transmitted helminths are a group of parasitic worms responsible for extensive morbidity in many of the world’s most economically depressed locations. With growing emphasis on disease mapping and eradication, the availability of accurate and cost-effective diagnostic measures is of paramount importance to global control and elimination efforts. While real-time PCR-based molecular detection assays have shown great promise, to date, these assays have utilized sub-optimal targets. By performing next-generation sequencing-based repeat analyses, we have identified high copy-number, non-coding DNA sequences from a series of soil transmitted pathogens. We have used these repetitive DNA elements as targets in the development of novel, multi-parallel, PCR-based diagnostic assays.

**Methodology/Principal Findings:**

Utilizing next-generation sequencing and the Galaxy-based RepeatExplorer web server, we performed repeat DNA analysis on five species of soil transmitted helminths (*Necator americanus*, *Ancylostoma duodenale*, *Trichuris trichiura*, *Ascaris lumbricoides*, and *Strongyloides stercoralis*). Employing high copy-number, non-coding repeat DNA sequences as targets, novel real-time PCR assays were designed, and assays were tested against established molecular detection methods. Each assay provided consistent detection of genomic DNA at quantities of 2 fg or less, demonstrated species-specificity, and showed an improved limit of detection over the existing, proven PCR-based assay.

**Conclusions/Significance:**

The utilization of next-generation sequencing-based repeat DNA analysis methodologies for the identification of molecular diagnostic targets has the ability to improve assay species-specificity and limits of detection. By exploiting such high copy-number repeat sequences, the assays described here will facilitate soil transmitted helminth diagnostic efforts. We recommend similar analyses when designing PCR-based diagnostic tests for the detection of other eukaryotic pathogens.

## Author Summary

With a growing emphasis on the mapping and elimination of soil transmitted helminth (STH) infections, the need for optimal and specific diagnostic methods is increasing. While PCR-based diagnostic methods for the detection of these parasitic organisms exist, these assays make use of sub-optimal target sequences. By designing assays that target non-coding, high copy-number repetitive sequences, both the limit of detection and species-specificity of detection improve. Using next-generation sequencing technology, we have identified high copy-number repeats for a series of STH species responsible for the greatest burden of disease. Using these repetitive sequences as targets in the design of novel real-time PCR assays, we have improved both the limits of detection and species-specificity of detection, and we have demonstrated this improved detection by testing these assays against an established PCR-based diagnostic methodology. Accordingly, these assays should facilitate mapping and monitoring efforts, and the generalized application of this approach to assay design should improve detection efforts for other eukaryotic pathogens.

## Introduction

Estimated to infect more than one quarter of the world’s total population, the soil transmitted helminths (STH) are responsible for profound morbidity and nutritional insufficiency [1]. Concentrated in the world’s most impoverished locations, the results of widespread infection on economic capacity are equally burdensome. Yet despite the scope of such disease, and continuing efforts to improve treatment programs and integration strategies, reliable and accurate diagnosis of STH infections remains difficult, and resulting prevalence estimates remain imprecise [1–2].

In recent years, the interest in molecular diagnostic methods for the detection of gastrointestinal helminths has grown substantially. Largely, this escalation in interest has occurred in parallel with the belief that standard microscopy-based methodologies for the examination of stool samples are sub-optimal, leading to underrepresentation of infection [3–5]. Further complicating matters, rates of STH egg/larval excretion have been shown to vary considerably within sequentially collected stool samples originating from a single infected individual [6–7]. This variability in egg/larval count can result in false negative samples, particularly when non-amplification-based diagnostic methodologies are utilized [7]. Such underrepresentation of disease complicates programmatic efforts, making the accurate assessment of the effects of intervention difficult, and frequently leaving low-level infections undiagnosed [5, 8–9]. Additionally, microscopy-based diagnostic methods have been linked with pathogen misidentification due to the morphological similarities that exist between species [5, 10]. Because of such concerns, a number of conventional and real-time PCR-based assays have been developed with the objective of improving both species-specificity and limits of detection [4, 11–17]. These assays have proven valuable, and as global efforts to estimate the burden of disease caused by the soil transmitted helminths (STHs) continue to increase, the number of studies incorporating such assays has risen in response [3, 5, 9, 18–21]. To date, the target sequences for such assays have been ribosomal internal transcribed spacer (ITS) sequences, 18S or ribosomal subunit sequences, or mitochondrial genes such as cytochrome oxidase I (COI) [4, 11–14]. Ribosomal sequences have been selected as diagnostic targets because they are typically found as easily identified moderate copy number tandem repeats in nucleated organisms [22–25]. Similarly, multiple copies of mitochondrial targets are found in the vast majority of eukaryotic cells [26], making them attractive target choices. However, while effective, such diagnostic targets are often sub-optimal. This is particularly true in the case of nematodes and other multi-cellular organisms where species-specific, highly repetitive DNA elements frequently make up a substantial portion of the genome, and are often present at copy-numbers exceeding 1,000 per haploid genome [27–29]. Due to such overrepresentation, non-coding repetitive sequence elements have become the targets of choice for many PCR-based diagnostic assays for the detection of various helminth species [30–31]. However, the identification of such repeats has historically been complicated and labor intensive. This identification has relied on techniques such as restriction endonuclease digestion of genomic DNA, followed by gel electrophoresis and Sanger DNA sequencing or polyacrylamide slab gel sequencing [32–34]. However, the advent of next-generation sequencing (NGS) technologies and associated informatics tools has expedited the search for highly repetitive sequence elements [35–39], and greater confidence can be placed in the accuracy of the results of such searches. Furthermore, as ribosomal and mitochondrial sequences tend to demonstrate high degrees of conservation between species, species-specificity of detection is also improved through the targeting of unique, highly-divergent, non-coding repeat DNA elements.

Here we describe the development of a multi-parallel real-time PCR assay for the detection of five species of soil transmitted helminths (*Necator americanus*, *Ancylostoma duodenale*, *Trichuris trichiura*, *Strongyloides stercoralis*, and *Ascaris lumbricoides*). Using NGS-generated sequence data and the Galaxy-based RepeatExplorer computational pipeline [38–39], we have searched the genomes of each organism for highly repetitive, non-coding DNA elements in order to identify diagnostic targets capable of providing optimal limits of detection and species-specificity of detection. Using these targets to design small-volume, multi-parallel tests [4], we have created a platform that provides cost-minimizing implementation of only those assays appropriate for a specific geographic region based upon the infections present. While performing multiplex assays may provide labor and time savings in locations where many parasites are co-endemic, such assays result in considerable waste when used in areas harboring only one or a few of the target species. In such settings, the “pick-and-choose” nature of multi-parallel assays minimizes reagent waste, and by improving upon limits of detection, the species-specific platform we describe here should facilitate improved STH monitoring and mapping efforts. Since NGS-based repeat analyses allow for the selection of the most efficacious target sequences, this approach to assay design should be applied to the development of additional diagnostics tests for other eukaryotic pathogens.

## Materials and Methods

### Isolation of parasite genomic DNA

For isolation of genomic DNA from *N*. *americanus*, *A*. *duodenale*, and *T*. *trichiura*, extractions were performed on cryopreserved adult worms in accordance with the “SWDNA1” protocol available on the Filarial Research Reagent Resource Center website (http://www.filariasiscenter.org/parasite-resources/Protocols/materials-1/). For *N*. *americanus* and *A*. *duodenale*, DNA extractions were conducted using a pool of approximately 10 adult worms. Both hookworm species belonged to strains originating in China. In the case of *T*. *trichiura*, extraction was performed using a single adult female worm of Ugandan origin. For *S*. *stercoralis* and *A*. *lumbricoides*, previously extracted genomic DNA was received from collaborators. *S*. *stercoralis* DNA was obtained from laboratory-reared worms originating from Pennsylvania, USA, and *A*. *lumbricoides* DNA was isolated from worms obtained from Ecuador.

### Next-generation sequencing of genomic DNA

#### Library preparation

50 ng of genomic DNA, at a concentration of 2.5 ng/μl, was utilized for the NGS library preparation of all organisms except *S*. *stercoralis*. For *S*. *stercoralis*, sequencing was not performed, as publically available sequence reads were used for the bioinformatics analyses (Sequence Read Archive ID: ERX044031). For all remaining parasites, libraries were prepared using the Nextera DNA Sample Preparation Kit (Illumina, San Diego, CA), the Nextera DNA Sample Preparation Index Kit (Illumina), and the ZR-96 DNA Clean & Concentrator-5 Kit (Zymo Research Corporation, Irvine, CA) in accordance with the manufacturer’s protocols and previous description [40]. Following library preparation, the concentration of each library was determined using the Qubit 1.0 Fluorometer (Life Technologies, Carlsbad, CA) and the Qubit dsDNA Broad Range Assay Kit (Life Technologies). Additionally, the size distribution of each library was analyzed using the Agilent 2100 Bioanalyzer System (Agilent Technologies, Santa Clara, CA) and the Agilent High Sensitivity DNA Kit (Agilent Technologies).

#### Next-generation sequencing

Based upon the DNA concentrations and size distributions for each prepared library, aliquots containing approximately 12 pmol of library were created for all parasites. Library aliquots were then sequenced individually on the MiSeq platform (Illumina) using the MiSeq Reagent Kit v3 (150 cycles) (Illumina) and the single-ended read approach.

### Repeat analysis

**Fig 1:**
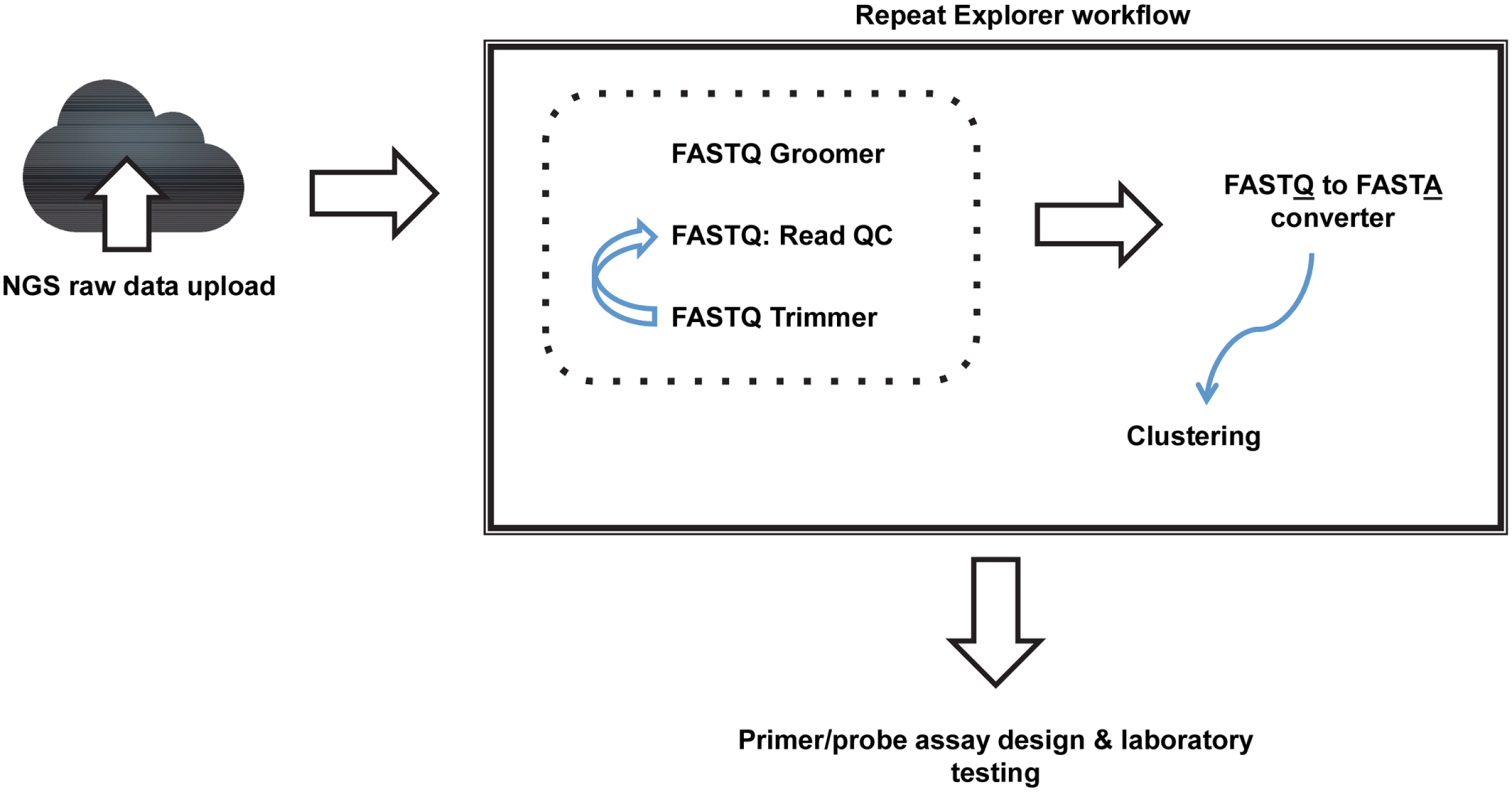
Workflow for repeat analysis. Output data from a next-generation sequencing run are uploaded to the RepeatExplorer Galaxy-based platform. During the QC and manipulation phase, the *FASTQ Groomer* tool is used to convert sequence reads into Sanger format. The *FASTQ*: *READ QC* tool is then used to verify the quality of the reads before removing unnecessary sequence (i.e. adapter sequences, etc.) from the ends of each read using the *FASTQ Trimmer* tool. The QC analysis is then repeated, and the *FASTQ to FASTA converter* tool is used to convert each read into FASTA format. Using these DNA sequence reads as input, sequences undergo clustering, during which an “all-to-all” sequence comparison is performed, and similar sequences are grouped together into clusters. Clusters containing the most highly repetitive sequences are then selected as putative diagnostic targets to be used for primer and probe-based real-time PCR assay design.

**Fig 2:**
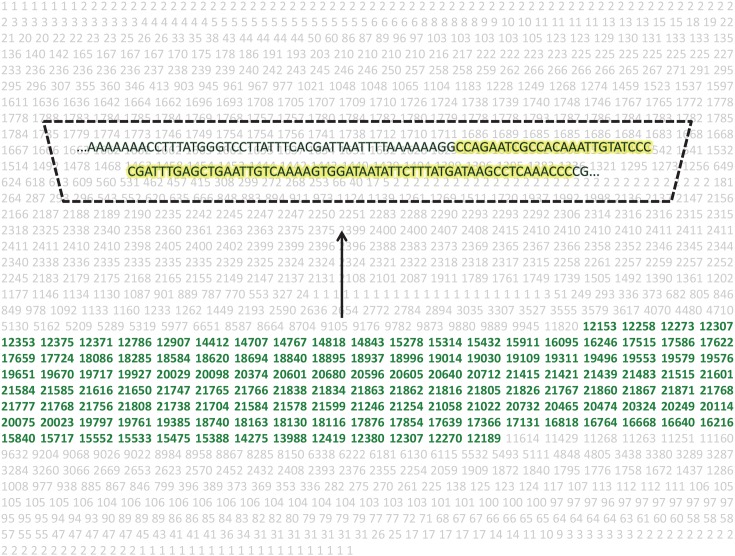
Illustrative output from RepeatExplorer analysis of *Necator americanus*. During “clustering” each nucleotide within a cluster is assigned a number. That number corresponds to how many individual next-generation sequencing reads that particular nucleotide appeared in. Using this output, a stretch of the most abundant nucleotides (depicted in green within the larger cluster’s sequence) is selected, and the corresponding nucleotides (highlighted in yellow) are selected as the candidate sequence from which the primers and probe are designed.

For each parasite analyzed, raw sequencing reads were uploaded to the Galaxy-based RepeatExplorer web server [39]. Reads were processed according to the workflow in Fig 1, enabling the identification of high copy-number repeat DNA sequences for each organism. Promising repeat families were further analyzed using the Nucleotide BLAST tool (http://blast.ncbi.nlm.nih.gov/Blast.cgi) available from the National Center for Biotechnology Information (NCBI). Results from each organism were screened for repetitive DNA elements found to have high degrees of homology with elements of the human genome, common bacteria of the human microbiome, or other parasitic organisms likely to be found within the human gut. Had such sequences been identified as among the most repetitive, they would have been eliminated from further consideration as they would be expected to cause species-specificity challenges during downstream PCR assay development. However, no such conserved highly repetitive elements were identified. Following screening, sequences from each organism, putatively determined to be among the most highly repetitive, were utilized for further assay development (Fig 2).

### Primer and probe design

**Fig 3:**
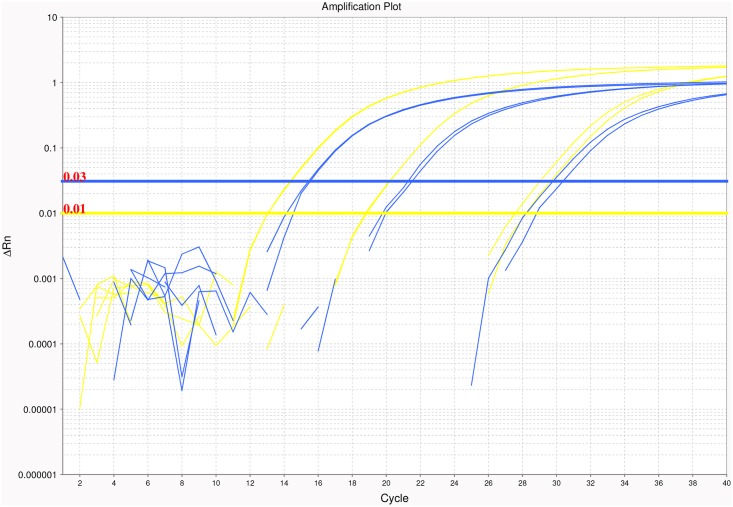
Comparative probe testing. For each novel probe design, FAM-TAMRA and double quenched FAM-ZEN-IOWA BLACK probes were synthesized. Comparative testing revealed that double quenched probes outperformed traditional probes, as evidenced by lower Ct values and greater ΔRn values. The plot above demonstrates these findings with the amplification of three concentrations of *N*. *americanus* template DNA using both double quenched (yellow) and traditional (blue) probe designs.

Candidate primer and probe pairings for each organism, excluding *A*. *lumbricoides*, were designed using the PrimerQuest online tool (Integrated DNA Technologies, Coralville, IA), utilizing the default parameters for probe-based qPCR. The putative species-specificity of each primer pair was further examined using Primer-BLAST software (http://blast.ncbi.nlm.nih.gov/Blast.cgi). In the case of *S*. *stercoralis* the highest copy-number repeat (as determined by RepeatExplorer) was not selected as a target sequence, due to design difficulties associated with the extreme A-T richness of the repeat (A-T % = 80.25). As a result, a second repeat analysis was performed, selecting only for sequence reads with > 30% G-C content, and a second candidate sequence was selected based on these results. In the case of *A*. *lumbricoides*, RepeatExplorer analyses of two different sequencing runs performed from two distinct libraries both resulted in the identification of ribosomal and mitochondrial sequences as the most highly repetitive. For this reason, sequences from an existing, proven, primer and probe set targeting the ITS1 region were selected for further analysis [14, 16]. With the exception of the previously published *A*. *lumbricoides* probe, all probes were labeled with a 6FAM fluorophore at the 5’ end, and were double quenched using the internal quencher ZEN and 3IABkFQ (IOWA BLACK) at the 3’ end (Integrated DNA Technologies). This fluorophore-quencher combination was chosen as comparative testing of each probe revealed improved Ct values and greater ΔRn values using this chemistry when compared to typical TAMRA quenching (Fig 3). Primer and probe sets for each organism can be found in Table 1.

**Table 1:** Selected primer and probe sequences for each multi-parallel assay.

Parasite	Forward Primer	Reverse Primer	Probe
***Necator americanus***	5’- CCAGAATCGCCACAAATTGTAT -3’	5’- GGGTTTGAGGCTTATCATAAAGAA -3’	5'- /56-FAM/CCCGATTT G/ZEN/AGCTGAATTGTCA AA/3IABkFQ/ -3'
***Ancylostoma duodenale***	5’- GTATTTCACTCATATGATCGAGTGTTC -3’	5’- GTTTGAATTTGAGGTATTTCGACCA -3’	5'- /56-FAM/TGACAGTG T/ZEN/GTCATACTGTGGA AA/3IABkFQ/ -3'
***Trichuris trichiura***	5’- GGCGTAGAGGAGCGATTT -3’	5’- TACTACCCATCACACATTAGCC -3’	5'- /56-FAM/TTTGCGGGC/ZEN/G AGAACGGAAATA TT/3IABkFQ/ -3'
***Strongyloides stercoralis***	5’- CGCTCCAGAATTAGTTCCAGTT -3’	5’- GCAGCTTAGTCGAAAGCATAGA -3’	5'- /56-FAM/ACAGTCTC C/ZEN/AGTTCACTCCAGA AGAGT/3IABkFQ/ -3'
***Ascaris lumbricoides***	5’- GTAATAGCAGTCGGCGGTTTCTT -3’	5’- GCCCAACATGCCACCTATTC -3’	5’- /56-FAM/ TTGGCGGACAATTGCATGCGAT/MBG/ -3’

### Primer and probe validation

#### Primer optimization reactions

In order to determine the optimal primer concentrations for each assay, a concentration matrix was created. For all primers, testing at 62.5 nM, 125 nM, 250 nM, 500 nM and 1000 nM was performed, and all forward primer concentrations were tested in combination with all reverse primer concentrations. Optimization assays were conducted in 7 μl volumes, containing 3.5 μl of 2X TaqMan Fast Universal PCR Master Mix (Life Technologies), 125 nmol of each assay’s respective probe, and 2 μl of template DNA at a concentration of 1 ng/μl. Cycling conditions consisted of an initial 2 min incubation step at 50°C, followed by a 10 min incubation at 95°C. These incubations were followed by 40 cycles of 95°C for 15 sec for denaturation, followed by 1 min at 59°C for annealing and extension. All reactions were conducted using the StepOne Plus Real-Time PCR System (Life Technologies).

#### Determination of assay detection limits

In order to determine the limits of detection for each assay, genomic template DNA stocks were titrated for each parasite. DNA stock concentrations of 1 ng/μl, 100 pg/μl, 10 pg/μl, 1 pg/μl, 100 fg/μl, 10 fg/μl, 1 fg/μl, 100 ag/μl, 10 ag/μl and 1 ag/μl were tested with each assay using the optimized primer concentrations and assays were again conducted in 7 μl total volumes. Reagent concentrations and cycling conditions were identical to those used for primer optimization reactions.

#### Assay species-specificity testing

In order to ensure the species-specificity of each assay, the primer-probe set for each parasite was tested using template DNA from each of the other STH species. Furthermore, each primer-probe combination was tested against human genomic DNA and the DNA of the common gastrointestinal tract commensal *Escherichia coli* (K-12 strain). All template stocks were at a concentration of 1 ng/μl, and all assays were performed using the same reagent volumes and concentrations as used for the primer optimization reactions and for the determination of assay detection limits.

### Comparative testing of field-collected samples

#### Collection of samples

For comparative assay testing, a panel of 79 samples was employed. All samples had been previously collected as part of the “Wash for Worms” interventional trial in Timor-Leste (Trial registration: ACTRN12614000680662). The specific procedures used during the collection and storage of these samples have been previously described [41].

#### DNA extraction

All DNA extractions were performed at QIMR Berghofer using the PowerSoil DNA isolation Kit (Mo Bio, Carlsbad, CA, USA) in accordance with the previously described, modified version of the manufacturer’s protocol [42]. Following extraction, an aliquot of each sample was retained at QIMR Berghofer and another was shipped to Smith College (Northampton, MA, USA).

#### Real-time PCR testing

DNA extracts from all samples were assayed at both QIMR Berghofer and Smith College. Testing at QIMR Berghofer was conducted using previously described real-time PCR primer/probe sets [4, 13–15], optimized for use in pentaplex assays [42] to test for the presence of *N*. *americanus*, *T*. *trichiura*, *Ascaris* ssp., *Ancylostoma* ssp., and *S*. *stercoralis*. For ease of reporting, hereafter, these assays will be referred to as the “QIMR assays”. Testing which occurred at Smith College made use of the optimized, previously undescribed multi-parallel assays for the detection of *N*. *americanus*, *T*. *trichiura*, *A*. *lumbricoides*, *A*. *duodenale*, and *S*. *stercoralis* introduced in this manuscript (hereafter referred to as the “Smith assays”). All sample aliquots tested at Smith College were coded blind by QIMR and assay results were not shared between institutions until all testing had been completed.

#### Statistical analysis

Positive, negative and overall agreements were calculated to assess concordance between the QIMR and Smith assays using the equations found in “Statistical Guidance on Reporting Results from Studies Evaluating Diagnostic Tests” [43]. Kappa statistics, which account for the possibility that concordance may occur by chance, were calculated for each taxon using R version 3.1.3 and the R package irr [44–45].

#### *Trichuris* speciation of Smith-negative, QIMR-positive samples

Samples testing positive for *Trichuris* ssp. using the QIMR assay, but negative for *Trichuris trichiura* using the Smith assay were further analyzed to determine the identity of the infecting species. Using a previously described primer-probe set targeting the coding sequence for the 18S ribosomal subunit [46], these samples were PCR amplified in 25 μl reactions using the Phusion Hot Start Flex DNA Polymerase Kit (New England Biolabs, Ipswich, MA). PCR reaction conditions were as follows: 16.5 μl PCR-grade water, 400 nM forward primer, 400 nM reverse primer, 0.5 μl dNTPs, 0.75 μl DMSO, 5.0 μl Phusion HF buffer, 0.25 μl Phusion Polymerase, and 1 μl template DNA. Cycling conditions consisted of an initial denaturing step at 98°C for 15 min, followed by 35 cycles of 98°C for 10 sec, 56°C for 15 sec, and 72°C for 15 sec. Following 35 cycles, a final extension step of 72°C for 7 min was performed. PCR products were then sequenced using standard Sanger methodology and resulting sequence data were analyzed using NCBI’s Nucleotide BLAST tool.

#### Differentiation of *A*. *duodenale* and *A*. *ceylanicum* infections

Samples testing positive for *Ancylostoma* ssp. using the QIMR assay, but negative for *A*. *duodenale* using the Smith assay, underwent further testing to discriminate between infection with *A*. *duodenale* and *A*. *ceylanicum*. For differential detection, a semi-nested PCR-Restriction Fragment Length Polymorphism (RFLP) assay was employed, and samples were tested in accordance with the published protocol [47]. Digestion of *Ancylostoma* ssp. PCR product using the MvaI enzyme (Life Technologies) was indicative of the presence of *A*. *ceylanicum*, while digestion with Psp1406I (Life Technologies) was indicative of *A*. *duodenale*.

## Results

### Primer and probe validation

#### Primer optimization

The use of a primer matrix resulted in the determination of optimal concentrations for each assay. Optimal conditions were determined to be those at which Ct values were lowest when testing 2 μl of the appropriate template stock at a concentration of 1 ng/μl. For *N*. *americanus*, *A*. *duodenale*, and *S*. *stercoralis*, the optimal concentrations were determined to be 250 nM for both forward and reverse primers. For *A*. *lumbricoides* the optimal concentrations were determined to be 62.5 nM for both forward and reverse primers, and for *T*. *trichiura*, the optimal concentrations were determined to be 62.5 nM for the forward primer and 250 nM for the reverse primer.

#### Assay sensitivities

In order to demonstrate the detection limits of each multi-parallel PCR assay, genomic DNA stocks for each parasite were serially diluted, and optimal primer concentrations for each assay were used to test 2 μl of the appropriate template at concentrations of 1 ng/μl, 100 pg/μl, 10 pg/μl, 1 pg/μl, 100 fg/μl, 10 fg/μl, 1 fg/μl, 100 ag/μl, 10 ag/μl, and 1 ag/μl. For all species, consistent detection of parasite DNA was possible at all concentrations at or above 1 fg/μl. For the detection of *A*. *duodenale*, consistent detection occurred at all concentrations at or above 10 ag/μl, and for the detection of *N*. *americanus*, sporadic detection proved possible at the 100 ag/μl and 10 ag/μl concentrations.

#### Assay specificities

To verify that the primer-probe combinations for the detection of each parasite were species-specific, each optimized assay was tested against genomic template DNA from each of the other parasite species included within this multi-parallel platform. They were also tested against human genomic DNA and *E*. *coli* genomic DNA. In no instance did species-specificity testing result in the amplification of any non-target DNA template, indicating that each assay demonstrated excellent species specificity.

### Comparative testing of field collected samples

A panel of 79 blindly-coded patient samples, obtained in Timor-Leste as part of a previously described study [42], was tested using the newly described multi-parallel Smith assays, as well as the previously described, multiplex real-time PCR detection methodology (QIMR assay) (Table 2, S1 Table). As samples were patient-obtained and no true “gold standard” exists for the detection of the various STH infections examined here, it is difficult to definitively determine whether increased sample positivity is a result of improved assay detection limits or non-specific, off-target amplification. For this reason, the comparative performances of each assay were assessed through calculations of positive, negative, and overall agreement [43]. For the detection of *N*. *americanus*, a positive agreement (PA) of 100% and a negative agreement (NA) of 61% were calculated. This resulted in an overall agreement (PO) of 85% (Kappa 0.658). Use of the Smith assay resulted in the detection of 60 positive samples, while the QIMR assay resulted in the detection of 48 positives. All 48 QIMR-positive samples were among the 60 positive samples detected using the Smith methodology.

**Table 2:** Comparative assay results for each species of parasite.

***Necator americanus***	*Smith-positive*	*Smith-negative*
*QIMR-positive*	48	0
*QIMR-negative*	12	19
***Ascaris lumbricoides***	*Smith-positive*	*Smith-negative*
*QIMR-positive*	40	0
*QIMR-negative*	7	32
***Trichuris trichiura***	*Smith-positive*	*Smith-negative*
*QIMR-positive*	10	4*
*QIMR-negative*	8	57
***Strongyloides stercoralis***	*Smith-positive*	*Smith-negative*
*QIMR-positive*	1	0
*QIMR-negative*	0	78
***Ancylostoma duodenale***	*Smith-positive*	*Smith-negative*
*QIMR-positive*	0	22 **
*QIMR-negative*	0	57

* Two of these four samples contained *T*. *ovis*. The identity of the pathogen in the remaining 2 samples could not be determined due to a lack of material.

** 21 of these 22 samples contained *A*. *ceylanicum*. The identity of the pathogen in the 22^nd^ sample could not be determined due to a lack of material.

For the detection of *A*. *lumbricoides*, a PA of 100%, an NA of 82%, and a PO of 91% (Kappa 0.822) were seen. The Smith assay for *A*. *lumbricoides* detection resulted in the identification of 47 positive samples, while the corresponding QIMR assay resulted in 40 positives. Again, all 40 QIMR-positive samples were among the 47 Smith-positive samples which were identified.

Detection of *Trichuris* gave a PA of 71%, an NA of 88% and a PO of 85% (Kappa 0.580). Sample examination using the Smith assay identified 18 positive extracts, while examination with the QIMR assay identified 14 positives. However, only 10 positives were common to both assays, with 8 samples identified as positive only by the Smith assay, and 4 samples demonstrating the presence of parasite DNA using only the QIMR methodology. Amplification in control reactions demonstrated that the QIMR assay, but not the Smith assay, would provide for the detection of the closely related parasite *Trichuris vulpis*, a whipworm species common to canines, but also known to cause zoonotic infection [48–49]. As *Trichuris* ssp. including *T*. *vulpis*, *Trichuris suis*, and *Trichuris ovis* have a wide geographic distribution with increased prevalence in tropical and sub-tropical locations [50–51], the four QIMR-positive, Smith-negative samples were sequenced to determine the identity of the *Trichuris* species present within these samples. BLAST analysis indicated that two of the samples contained DNA from the ruminant parasite *T*. *ovis* (E values = 0.0). Unfortunately, two independent trials failed to produce usable sequence for the remaining two samples, after which both sample stocks had been exhausted, making further examination impossible.

Examination of all 79 samples for the presence of *S*. *stercoralis* resulted in the detection of only a single positive sample. This single sample was identified using both the Smith and QIMR assays. Sample examination for the presence of *Ancylostoma* resulted in the identification of 22 *Ancylostoma* ssp. positive samples using the QIMR methodology. However, not a single *A*. *duodenale*-positive sample was identified using the Smith assay. As the zoonotic parasite *Ancylostoma ceylanicum* has been suspected of causing human infection in Timor-Leste [52], a previously described, semi-nested PCR-RFLP assay was employed to discriminate infection with *A*. *duodenale* from infection with *A*. *ceylanicum* [47]. In this assay, an MvaI restriction digest of PCR product is indicative of the presence of *A*. *ceylanicum*, while digestion with Psp1406I is indicative of *A*. *duodenale*. 21 of the 22 *Ancylostoma* ssp. positive samples were digested by MvaI, identifying the infections as *A*. *ceylanicum* in origin. Two independent PCR trials (four replicates) failed to amplify the remaining *Ancylostoma* ssp.-positive sample, preventing a definitive determination of the identity of the parasite in that sample.

Because a sizeable panel of field-collected samples was analyzed using the two different real-time PCR methodologies discussed here, a comparison of Ct values was conducted for all samples testing positive for a given parasite by both the Smith and QIMR methods (S1 Table). All 10 samples demonstrating positive results for *T*. *trichiura* when tested by both assays showed lower Ct values using the Smith methodology (mean difference in Ct value = 7.86 +/- 2.46). Examination for *N*. *americanus* resulted in a similar pattern, with all 48 samples testing positive by both methodologies possessing lower Ct values when tested using the Smith assay (mean difference in Ct value = 4.94 +/- 1.22). In the case of *A*. *lumbricoides*, Ct values were lower using the QIMR methodology for 38 of 40 samples demonstrating positive results for both assays. However, at 0.896 +/- 0.767, the mean difference in Ct values was low. For *S*. *stercoralis* testing, only a single positive sample was identified. This sample possessed a lower Ct value when tested using the Smith assay. As no samples tested positive for *Ancylostoma* using the Smith assay (QIMR-positive samples were demonstrated to be *A*. *ceylanicum*), a Ct comparison could not be made.

## Discussion

In light of their impact on global health, the importance of optimal and species-specific diagnostic methods for the detection of soil transmitted helminths cannot be overestimated. While current molecular assays making use of ribosomal and mitochondrial targets have vastly improved the diagnosis of STH infection, these targets are frequently sub-optimal, potentially leaving low-level infections undiagnosed. Furthermore, such sequences may lack the species-specificity required to discriminate between different species of the same genus. In contrast, assays targeting high copy-number repetitive sequences improve upon assay detection limits, as many eukaryotic pathogens contain large numbers of such non-coding repeat DNA elements. Accordingly, by coupling the high throughput nature of NGS with the Galaxy-based RepeatExplorer computational pipeline, a cost effective, accurate, and expedited methodology for the identification of high copy-number repeat DNA elements was developed. Through the design of real-time PCR primer/probe pairings that uniquely target such repetitive sequences in a species-specific manner, diagnostic accuracy and limits of detection are improved dramatically when compared with microscopy-based diagnostic techniques and PCR-based diagnostics targeting mitochondrial or ribosomal sequences. Utilizing this strategy, we have successfully identified novel target sequences for the detection of *N*. *americanus*, *A*. *duodenale*, *T*. *trichiura*, and *S*. *stercoralis*. Furthermore, we have demonstrated the consistent detection of genomic DNA from each target organism at quantities of 2 fg or less, and have presented evidence to suggest improved limits of detection and species-specificity relative to an established and validated PCR diagnostic methodology [Llewellyn, 2016]. Although further testing utilizing “spiked” samples containing known quantities of eggs/larvae is currently underway, 2 fg of DNA is far less than the quantity present within a single fertilized egg or L1 larvae of each species [53–55] (Table 3). In principle, we have therefore demonstrated the potential of these assays to detect a single egg within a tested patient stool sample.

**Table 3:** Estimated Minimum Quantities of DNA in the Eggs of Each Species of STH.

Species	Haploid Genome Size (Genbank Assembly Accession)	Estimated Minimum Quantity of DNA/Diploid Cell	Estimated Number of Diploid Cells/Egg^[^^55^^]^	Estimated Total Quantity of DNA/Egg
*Necator americanus*	244 Mb^[^^54^^]^ (GCA_000507365.1)	0.54 pg	4–8	2.16 pg– 4.32 pg
*Ascaris lumbricoides*	317 Mb (GCA_000951055.1)	0.70 pg	As few as 1	0.70 pg
*Trichuris trichiura*	75 Mb (GCA_000613005.1)	0.17 pg	1	0.17 pg
*Ancylostoma duodenale*	332 Mb (GCA_000816745.1)	0.73 pg	4–8	2.92 pg– 5.84 pg
*Strongyloides stercoralis*	42–60 Mb^[^^53^^]^ (GCA_000947215.1)	0.09 pg– 0.13 pg	2–8*	0.18 pg– 1.04 pg*

* As *S*. *stercoralis* eggs typically hatch in the intestinal lumen, this parasite is released into the stool as rhabditiform larvae which possess an even greater number of cells. Therefore, a patient stool sample harboring a single larval worm will contain a much higher quantity of parasite DNA than the quantity listed here.

While the high copy-number nature of non-coding repetitive sequence elements makes them attractive diagnostic targets, such elements also frequently demonstrate rapid evolutionary divergence [56–57]. This divergence increases the diagnostic appeal of these sequences, as divergence reduces the risk for non-specific, off-target amplification, a characteristic essential for the development of species-specific PCR assays capable of discriminating between closely related organisms. Accordingly, while additional testing against genomic DNA from a growing panel of closely related parasites will continue to be used to evaluate the species-specificity of each selected primer/probe set, we have successfully demonstrated that each Smith assay does not amplify off-target templates from any other parasite species included within this multi-parallel platform. Furthermore, by employing a semi-nested PCR-RFLP tool, we were able to successfully demonstrate that our assay for the detection of *A*. *duodenale* does not amplify the closely related parasite *A*. *ceylanicum*. In contrast, the previously published primer/probe set employed for comparative testing was unable to distinguish between these two species, resulting in consistent off-target amplification of *A*. *ceylanicum* DNA. Similarly, while our *T*. *trichiura* assay failed to amplify four samples containing genetic material from *Trichuris* ssp., the comparative QIMR assay again demonstrated non-specific, off-target amplification for at least two of these samples, as sequence analysis demonstrated the presence of DNA from the ruminant parasite *T*. *ovis*. Taken together, these findings support the notion that improved assay species-specificity results from non-coding, repeat-based PCR assay design. Of note, to our knowledge, this is the first example of *T*. *ovis* potentially serving as a causative agent of zoonotic infection. However, as sheep are considered a major agricultural commodity of Timor-Leste [58], the possibility exists that individuals testing positive for *T*. *ovis* may have ingested intestinal material from an animal harboring infection, making it conceivable that the *T*. *ovis* DNA present was not the result of zoonotic infection. Given that *T*. *ovis* is not known to cause human infection, further exploration of this possible zoonosis is warranted.

Attempting to design a non-coding, repetitive DNA sequence-based assay for the species-specific detection of *A*. *lumbricoides* presented a unique set of challenges. *A*. *lumbricoides*, like many species of Ascaridae, discards large portions of its highly repetitive, non-coding genomic DNA during embryonic development. This process, known as chromosome diminution, eliminates the presence of such DNA elements from post-embryonic somatic cells [59–61]. Presumably for this reason, two separate repeat analyses, performed on two distinct library preparations, failed to identify any repetitive sequences with copy numbers greater than ribosomal and mitochondrial targets. Accordingly, a previously described primer/probe set targeting the ITS1 ribosomal region was chosen for inclusion in our multi-parallel platform [14, 16]. In order to improve diagnostics for this parasite, further analysis of *A*. *lumbricoides* using DNA extracted from eggs alone (before chromosome diminution) will be undertaken.

In addition to the potential detection limit improvements and species-specificity gains realized when diagnostically targeting non-coding repetitive DNA sequences, designing multi-parallel assays provides another unique set of advantages over previous design strategies [4]. By reducing the number of tests required, multiplex assays can provide labor and reagent savings over alternative diagnostic measures when used in environments that harbor the full complement of organisms targeted by the assay [62–63]. However, as the geographic distribution of STH species is not uniform, the use of multi-parallel assays makes it possible to select only the assays appropriate for a given location, reducing primer/probe costs associated with testing for unnecessary targets [4]. By running these assays as “small-volume” 7 μl reactions, reagent use is minimized, resulting in cost savings. Furthermore, as multi-parallel reactions are run independently, this enables the development of new assays for new pathogens and their subsequent addition to the testing platform without the complex re-optimization of assay conditions required for multiplex PCR assays.

While reagent costs associated with performing molecular diagnostic testing are higher than costs associated with conducting traditional microscopy-based diagnostics, expenses associated with molecular techniques are declining as improved reagents and enzymes have allowed reaction volumes to decrease, minimizing reagent needs [4, 64]. Furthermore, reagent improvements have increased the practicality of sample pooling, a practice already adopted by many tropical disease surveillance and diagnostic efforts [65–69]. Such pooling allows for cost-reducing high-throughput screening of stool samples [70–71]. Thus, while the total cost associated with performing a duplicate Kato-Katz thick smear under field conditions has been estimated at $2.06 [72] and we estimate the total cost associated with the duplicate testing a single stool sample using all five multi-parallel assays to be approximately $10, the pooling of as few as five samples would render small volume, multi-parallel PCR testing more cost effective than Kato-Katz testing. Furthermore, molecular diagnostic accuracy and reliability provide increased clarity of results [64], allowing for the implementation of more informed and effective treatment and control strategies. Such improvements in efficiency result in greater programmatic gains, drastically reducing long-term costs and expenses of control or elimination programs.

One profound shortcoming which hampers STH diagnostic development is the lack of a reliable gold standard for detection [8]. While still used in many clinical, mapping, and research efforts, microscopy-based methodologies are known to lack both adequate limits of detection and species-specificity of detection [3–5, 10, 64]. Similarly, while currently available molecular methods have greatly improved upon many of the shortcomings inherent to microscopy, the use of sub-optimal ribosomal or mitochondrial targets possessing relatively high degrees of conservation can result in both false-negative, and off-target, false-positive results. Thus, a gold standard of detection is sorely needed. Unfortunately, without a definitive method for assigning positive/negative status to an unknown sample, distinguishing improved limits of detection from false-positive amplification can be difficult. Nonetheless, comparative assay testing remains an important aspect of designing any diagnostic test. As such, we believe the evaluation of Timor-Leste patient samples presented in this paper provides strong evidence for improved limits of detection when utilizing the newly described Smith assays. While strain-specific genetic differences arising within divergent geographic isolates could present detection challenges, testing on a limited number of patient-derived samples from Argentina and Ethiopia aimed at providing evidence for the global applicability of these multi-parallel assays is currently underway. Additional studies to further validate these assays on a variety of geographic isolates will continue.

In all instances, and for all parasites excluding *Ancylostoma* and *Trichuris* (where off-target amplification of *A*. *ceylanicum* and *T*. *ovis* by the QIMR assay was demonstrated), each Timor-Leste patient sample that provided a positive QIMR assay result also demonstrated positivity with the corresponding Smith assay. Furthermore, all *N*. *americanus*, *T*. *trichiura*, and *S*. *stercoralis* samples that were positive by both assays exhibited lower Ct values for the Smith assay results. These findings strongly suggest improved limits of detection for the Smith assays, and support our contention that samples returning Smith assay positive results, but QIMR assay negative results, are likely low-level positives escaping detection by the sub-optimal PCR platform. This conclusion is further supported by the finding that the Smith assays do not show off-target amplification of any other STH parasites, human DNA or *E*. *coli* DNA.

As both the QIMR and Smith assays for the detection of *A*. *lumbricoides* make use of the same previously published primer/probe combination [14, 16], comparative assay testing for this parasite provided results which were more difficult to interpret. As increased reaction volumes are known to frequently improve detection limits for an assay, likely due to the large volume nature of the QIMR assay (25 μl vs. 7 μl for Smith), 38 of 40 samples returning positive results for both testing platforms demonstrated lower Ct values when examined using the QIMR method. Interestingly, despite this tendency for QIMR testing to result in lower Ct values, seven samples identified as positive using the Smith assay were found to be QIMR-negative. In contrast, not a single sample was found to be QIMR-positive and Smith-negative. As the QIMR assays are multiplexed, one explanation for this apparent contradiction is that the multiplex methodology failed to detect *A*. *lumbricoides* in a subset of samples that were positive for multiple STH parasites (S1 Table). Such failures are known to occur in multiplex reactions, particularly when primer concentrations are suboptimal, as reagents are utilized for the amplification of a more prevalent target, preventing the amplification of the lower copy-number target sequences within the sample [73]. Alternatively, while the results of our assay specificity testing present compelling evidence to the contrary, the possibility of false positive amplification cannot be definitively ruled out.

Non-coding repetitive DNA elements are found in nearly all eukaryotic organisms. Such sequences are typically highly divergent, and frequently exist in high copy-number. These characteristics make them ideal molecular diagnostic targets, particularly for the detection of pathogens such as the STHs, which remain an underdiagnosed, poorly mapped global health concern. By applying next-generation sequencing technology to the challenge of repeat DNA discovery, we have designed highly specific multi-parallel PCR assays with improved limits of detection over existing diagnostic platforms. We believe that these assays will greatly aid in the global efforts to map STH infection, facilitating accurate disease prevalence estimates. Furthermore, we intend to apply this approach to molecular target discovery of other parasitic organisms and NTDs, as optimal limits of detection and species-specificity of detection are vital to all diagnostic efforts. This is particularly true when implementing diagnostics in climates of decreasing disease prevalence. Accordingly, as NTD elimination efforts continue to progress, optimized assays will play an increasingly critical role in the detection of sporadic and focal infections and the monitoring for disease recrudescence.

## Supporting Information

S1 Table Comparative assay results for patient-obtained sample from Timor-Leste. All samples were tested in duplicate utilizing both real-time PCR assays (QIMR and Smith) and results are provided as mean Ct values.
(XLSX)

## References

[1] Hotez PJ, Bundy DAP, Beegle K, Brooker S, Drake L, de Silva N, et al Helminth Infections: Soil-transmitted Helminth Infections and Schistosomiasis In: Disease Control Priorities in Developing Countries. Washington: World Bank; 2006 pp. 467–482.

[2] Buonfrate D, Formenti F, Perandin F, Bisoffi Z. Novel approaches to the diagnosis of *Strongyloides stercoralis* infection. Clin Microbiol Infect. 2015; 21(6): 543–552. 10.1016/j.cmi.2015.04.001 25887711

[3] Schär F, Odermatt P, Khieu V, Panning M, Duong S, Muth S, et al Evaluation of real-time PCR for *Strongyloides stercoralis* and hookworm as diagnostic tool in asymptomatic schoolchildren in Cambodia. Acta Trop. 2013; 126(2): 89–92. 10.1016/j.actatropica.2012.12.012 23298731

[4] Mejia R, Vicuña Y, Broncano N, Sandoval C, Vaca M, Chico M, et al A novel, multi-parallel, real-time polymerase chain reaction approach for eight gastrointestinal parasites provides improved diagnostic capabilities to resource-limited at-risk populations. Am J Trop Med Hyg. 2013; 88(6): 1041–1047. 10.4269/ajtmh.12-0726 23509117 PMC3752800

[5] Gordon CA, McManus DP, Acosta LP, Olveda RM, Williams GM, Ross AG, et al Multiplex real-time PCR monitoring of intestinal helminthes in humans reveals widespread polyparasitism in Northern Samar, the Philippines. Int J Parasitol. 2015; 45(7): 477–483. 10.1016/j.ijpara.2015.02.011 25858090

[6] Anderson RM, Schad GA. Hookworm burdens and faecal egg counts: an analysis of the biological basis of variation. Trans R Soc Trop Med Hyg. 79(6): 812–825. 3832493 10.1016/0035-9203(85)90128-2

[7] Booth M, Vounatsou P, N’Goran EK, Tanner M, Utzinger J. The influence of sampling effort and the performance of the Kato-Katz technique in diagnosing *Schistosoma mansoni* and hookworm co-infections in rural Côte d’Ivoire. Parasitology. 2003; 127(Pt 6): 525–531. 14700188 10.1017/s0031182003004128

[8] Nikolay B, Brooker SJ, Pullan RL. Sensitivity of diagnostic tests for human soil-transmitted helminths infections: a meta-analysis in the absence of a true gold standard. Int J Parasitol. 2014; 44(11): 765–774. 10.1016/j.ijpara.2014.05.009 24992655 PMC4186778

[9] Knopp S, Salim N, Schindler T, Voules DAK, Rothen J, Lweno O, et al Diagnostic accuracy of Kato-Katz, FLOTAC, Baermann, and PCR Methods for the Detection of light-intensity hookworm and *Strongyloides stercoralis* Infections in Tanzania. Am J Trop Med Hyg. 2014; 90(3): 535–545. 10.4269/ajtmh.13-0268 24445211 PMC3945701

[10] Haque R, Mondal D, Karim A, Molla IH, Rahim A, Faruque ASG, et al Prospective case-control study of the association between common enteric protozoal parasites and diarrhea in Bangladesh. Clin Infect Dis. 2009; 48: 1191–1197. 10.1086/597580 19323634 PMC2883291

[11] Phuphisut O, Yoonuan T, Sanguankiat S, Chaisiri K, Maipanich W, Pubampen S, et al Triplex Polymerase Chain Reaction Assay for Detection of Major Soil-Transmitted Helminths, *Ascaris lumbricoides*, *Trichuris trichiura*, *Necator americanus*, in Fecal Samples. Southeast Asian J Trop Med Public Health. 2014; 45(2): 267–275. 24968666

[12] Basuni M, Muhi J, Nurulhasanah O, Verweij JJ, Ahmad M, Miswan N, et al A Pentaplex real-time polymerase chain reaction assay for detection of four species of soil-transmitted helminths. Am J Trop Med Hyg. 2011; 84(2): 338–343. 10.4269/ajtmh.2011.10-0499 21292911 PMC3029194

[13] Verweij JJ, Brienen EAT, Ziem J, Yelifari L, Polderman AM, Van Lieshout L. Simultaneous Detection and Quantification of *Ancylostoma duodenale*, *Necator americanus*, and *Oesophagostomum bifurcum* in Fecal Samples Using Multiplex Real-Time PCR. Am J Trop Med Hyg. 2007; 77(4): 685–690. 17978072

[14] Wiria AE, Prasetyani MA, Hamid F, Wammes LJ, Lell B, Ariawa I, et al Does treatment of intestinal helminth infections influence malaria? Background and methodology of a longitudinal study of clinical, parasitological and immunological parameters in Nangapanda, Flores, Indonesia (ImmunoSPIN Study). BMC Infect Dis. 2010; 10: 77 10.1186/1471-2334-10-77 20338054 PMC2859773

[15] Verweij JJ, Canales M, Polman K, Ziem J, Brienen EAT, Polderman AM, et al Molecular diagnosis of *Strongyloides stercoralis* in fecal samples using real-time PCR. Trans R Soc Trop Med Hyg. 2009; 103(4): 342–346. 10.1016/j.trstmh.2008.12.001 19195671

[16] Liu J, Gratz J, Amour C, Kibiki G, Becker S, Janaki L, et al A laboratory-developed TaqMan Array Card for simultaneous detection of 19 enteropathogens. J Clin Microbiol. 2013; 51(2): 472–480. 10.1128/JCM.02658-12 23175269 PMC3553916

[17] Janwan P, Intapan PM, Thanchomnang T, Lulitanond V, Anamnart W, Maleewong W. Rapid detection of *Opisthorchis viverrini* and *Strongyloides stercoralis* in human fecal samples using a duplex real-time PCR and melting curve analysis. Parasitol Res. 2011; 109(6): 1593–1601. 10.1007/s00436-011-2419-z 21537984

[18] Fabian S, Odermatt P, Khieu V, Panning M, Duong S, Muth S, et al Evaluation of real-time PCR for *Strongyloides stercoralis* and hookworm as diagnostic tool in asymptomatic schoolchildren in Cambodia. Acta Trop. 2013; 126(2): 89–92. 10.1016/j.actatropica.2012.12.012 23298731

[19] Cimino RO, Jeun R, Juarez M, Cajal PS, Vargas P, Echazú A, et al Identification of human intestinal parasites affecting an asymptomatic peri-urban Argentinian population using multi-parallel quantitative real-time polymerase chain reaction. Parasit Vectors. 2015; 8: 380 10.1186/s13071-015-0994-z 26183074 PMC4504406

[20] Tahapary DL, de Ruiter K, Martin I, van Lieshout L, Guigas B, Soewondo P, et al Helminth infections and type 2 diabetes: a cluster-randomized placebo controlled SUGARSPIN trial in Nangapanda, Flores, Indonesia. BMC Infect Dis. 2015; 15: 133 10.1186/s12879-015-0873-4 25888525 PMC4389675

[21] Staudacher O, Heimer J, Steiner F, Kayonga Y, Havugimana JM, Ignatius R, et al Soil-transmitted helminths in southern highland Rwanda: associated factors and effectiveness of school-based preventive chemotherapy. Trop Med Int Health. 2014; 19(7): 812–824. 10.1111/tmi.12321 24750543

[22] Gatehouse HS, Malone LA. The Ribosomal RNA Gene Region of *Nosema apis* (Microspora): DNA Sequence for Small and Large Subunit rRNA Genes and Evidence of a Large Tandem Repeat Unit Size. J Invertebr Pathol. 1998; 71(2): 97–105. 9547137 10.1006/jipa.1997.4737

[23] Hlinka O, Murrell A, Barker SC. Evolution of the secondary structure of the rRNA internal transcriber spacer 2 (ITS2) in hard ticks (Ixodidae, Arthropoda). Heredity. 2002; 88: 275–279. 11920135 10.1038/sj.hdy.6800040

[24] Gerbi SA. Evolution of ribosomal DNA In: Molecular Evolutionary Genetics. New York/London: Plenum Press; 1985 pp. 419–517.

[25] Fritz GN, Conn J, Cockburn A, Seawright J. Sequence Analysis of the Ribosomal DNA Internal Transcribed Spacer 2 from Populations of *Anopheles nuneztovari* (Diptera: Culicidae). Mol Biol Evol. 1994; 11(3): 406–416. 8015435 10.1093/oxfordjournals.molbev.a040122

[26] Alberts B, Johnson A, Lewis J, Raff M, Roberts K, Walter P. Molecular Biology of the Cell. 5th ed. New York: Garland Science; 2008.

[27] Cooper GM. The Cell: A Molecular Approach. 2nd Edition. Sunderland (MA): Sinauer Associates; 2000.

[28] The C. elegans Sequencing Consortium. Genome Sequence of the Nematode *C*. *elegans*: A Platform for Investigating Biology. Science. 1998; 282(5396): 2012–2018. 9851916 10.1126/science.282.5396.2012

[29] Thanchomnang T, Intapan PM, Lulitanond V, Choochote W, Manjai A, Prasongdee TK, et al Rapid detection of *Brugia malayi* in mosquito vectors using a real-time fluorescence resonance energy transfer PCR and melting curve analysis. Am J Trop Med Hyg. 2008; 78(3): 509–513. 18337351

[30] Alhassan A, Li Z, Poole CB, Carlow CK. Expanding the MDx toolbox for filarial diagnosis and surveillance. Trends Parasitol. 2015; 31(8): 391–400. 10.1016/j.pt.2015.04.006 25978936

[31] Ricciardi A, Ndao M. Diagnosis of Parasitic Infections: What’s Going On? J Biomol Screen. 2015; 20(1): 6–21. 10.1177/1087057114548065 25170017

[32] McReynolds LA, DeSimone SM, Williams SA. Cloning and comparison of repeated DNA sequences from the human filarial parasite *Brugia malayi* and the animal parasite *Brugia pahangi*. Proc Natl Acad Sci U.S.A. 1986; 83: 797–801. 3003750 10.1073/pnas.83.3.797 PMC322952

[33] Zhong M, McCarthy J, Bierwert L, Lizotte-Waniewski M, Chanteau S, Nutman TB, et al A polymerase chain reaction assay for detection of the parasite *Wuchereria bancrofti* in human blood samples. Am J Trop Med Hyg. 1996; 54(4): 357–363. 8615447 10.4269/ajtmh.1996.54.357

[34] Vargas N, Souto RP, Carranza JC, Vallejo GA, Zingales B. Amplification of a specific repetitive DNA sequence for *Trypanosoma rangeli* identification and its potential application in epidemiological investigations. Exp Parasitol. 2000; 96(3): 147–159. 11162365 10.1006/expr.2000.4563

[35] Treangen TJ, Salzberg SL. Repetitive DNA and next-generation sequencing: computational challenges and solutions. Nat Rev Genet. 2011; 13(1): 36–46. 10.1038/nrg3117 22124482 PMC3324860

[36] Subirana JA, Messeguer X. A Satellite Explosion in the Genome of Holocentric Nematodes. PLoS One. 2013; 8(4): e62221 10.1371/journal.pone.0062221 23638010 PMC3634726

[37] Roset R, Subirana JA, Messeguer X. MREPATT: detection and analysis of exact consecutive repeats in genomic sequences. Bioinformatics. 2003; 19(18): 2475–2476. 14668235 10.1093/bioinformatics/btg326

[38] Novák P, Neumann P, Macas J. Graph-based clustering and characterization of repetitive sequences in next-generation sequencing data. BMC Bioinformatics. 2010; 11:378 10.1186/1471-2105-11-378 20633259 PMC2912890

[39] Novák P, Neumann P, Pech J, Steinhaisl J, Macas J. RepeatExplorer: a Galaxy-based web server for genome-wide characterization of eukaryotic repetitive elements from next-generation sequence reads. Bioinformatics. 2013; 29(6): 792–793. 10.1093/bioinformatics/btt054 23376349

[40] Caruccio N. Preparation of next-generation sequencing libraries using Nextera technology: simultaneous DNA fragmentation and adapter tagging by in vitro transposition. Methods Mol Biol. 2011; 733: 241–55. 10.1007/978-1-61779-089-8_17 21431775

[41] Nery SV, McCarthy JS, Traub R, Andrews RM, Black J, Gray D, et al A cluster-randomized controlled trial integrating a community-based water, sanitation and hygiene programme with mass distribution of albendazole to reduce intestinal parasites in Timor-Leste: the WASH for WORMS research protocol. BMJ Open. 2015; 5(12): e009293 10.1136/bmjopen-2015-009293 26719316 PMC4710834

[42] Llewellyn S, Inpankaew T, Nery S, Gray DJ, Verweij JJ, Clements ACA, et al Application of a multiplex quantitative PCR to assess prevalence and intensity of intestinal parasite infections in a controlled clinical trial. PLoS Negl Trop Dis. 2016; 10(1): e0004380 10.1371/journal.pntd.0004380 26820626 PMC4731196

[43] U.S. Department of Health and Human Services Food and Drug Administration. Statistical guidance on reporting results from studies evaluating diagnostic tests. 2007. Available: http://www.fda.gov/MedicalDevices/DeviceRegulationandGuidance/GuidanceDocuments/ucm071148.htm.

[44] R Core Team. R: A language and environment for statistical computing. R Foundation for Statistical Computing, Vienna, Australia 2015 Available: https://www.r-project.org.

[45] Gamer M, Lemon J, Fellows I, Singh P. Package ‘irr’: Various Coefficients of Interrater Reliability and Agreement. 2012. Available: https://cran.r-project.org/web/packages/irr/irr.pdf.

[46] Meekums H, Hawash MB, Sparks AM, Oviedo Y, Sandoval C, Chico ME, et al A genetic analysis of *Trichuris trichiura* and *Trichuris suis* from Ecuador. Parasit Vectors. 2015; 8: 168 10.1186/s13071-015-0782-9 25889461 PMC4373032

[47] George S, Kaliappan SP, Kattula D, Roy S, Geldhof P, Kang G, et al Identification of *Ancylostoma ceylanicum* in Children from a tribal community in Tamil Nadu, India using a semi-nested PCR-RFLP tool. Trans R Soc Trop Med Hyg. 2015; 109(4): 283–285. 10.1093/trstmh/trv001 25618132

[48] Masuda Y, Kishimoto T, Ito H, Tsuji M. Visceral larva migrans caused by *Trichuris vulpis* presenting as a pulmonary mass. Thorax. 1987; 42(12): 990–991. 3438889 10.1136/thx.42.12.990 PMC461067

[49] Sakano T, Hamamoto K, Kobayashi Y, Sakata Y, Tsuji M, Usui T. Visceral larva migrans caused by *Trichuris vulpis*. Arch Dis Child. 1980; 55(8): 631–633. 7436519 10.1136/adc.55.8.631 PMC1627048

[50] Areekul P, Putaporntip C, Pattanawong U, Sitthicharoenchai P, Jongwutiwes S. *Trichuris vulpis* and *T*. *trichiura* infections among schoolchildren of a rural community in northwestern Thailand: the possible role of dogs in disease transmission. Asian Biomed. 2010; 4(1): 49–60.

[51] Hendrix CM, Robinson E. Diagnostic Parasitology for Veterinary Technicians. 4th ed. Philadelphia: Elsevier Health Sciences; 2014.

[52] Berger S. Infectious Diseases of East Timor. Los Angeles: GIDEON Informatics, Inc; 2015.

[53] Hunt VL, Tsai IJ, Coghlan A, Reid AJ, Holroyd N, Foth BJ, et al The genomic basis of parasitism in the *Strongyloides* clade of nematodes. Nat Genet. 2016; 10.1038/ng.3495 PMC4948059 26829753

[54] Tang YT, Gao X, Rosa BA, Abubucker S, Hallsworth-Pepin K, Martin J, et al Genome of the human hookworm *Necator americanus*. Nat Genet. 2014; 46(3): 261–269. 10.1038/ng.2875 24441737 PMC3978129

[55] Cuomo MJ, Noel LB, White DB. Diagnosing Medical Parasites: A Public Health Officers Guide to Assisting Laboratory and Medical Officers. USAF Air Education and Training Command, Randolph, TX: http://www.phsource.us/PH/PARA/; 2012.

[56] Meštrović N, Pavlek M, Car A, Castagnone-Sereno P, Abad P, Plohl M. Conserved DNA Motifs, Including the CENP-B Box-like, are Possible Promoters of Satellite DNA Array Rearrangements in Nematodes. PLoS One. 2013; 8(6): e67328 23826269 10.1371/journal.pone.0067328 PMC3694981

[57] Plohl M, Luchetti A, Mestrović N, Mantovani B. Satellite DNAs between selfishness and functionality: structure, genomics and evolution of tandem repeats in centromeric (hetero)chromatin. Gene. 2008; 409(1–2): 72–82. 10.1016/j.gene.2007.11.013 18182173

[58] da Cruz CJ. Livestock development in East Timor. In: Agriculture: New Directions for a New Nation—East Timor (Timor Leste). ACIAR Proceedings No. 113; 2003. pp. 11–16.

[59] Tobler H, Etter A, Müller F. Chromatin diminution in nematode development. Trends Genet. 1992; 8(12): 427–432. 1492368 10.1016/0168-9525(92)90326-y

[60] Goday C, Pimpinelli S. The occurrence, role and evolution of chromatin diminution in nematodes. Parasitol Today. 1993; 9(9): 319–322. 15463793 10.1016/0169-4758(93)90229-9

[61] Etter A, Bernard V, Kenzelmann M, Tobler H, Müller F. Ribosomal heterogeneity from chromatin diminution in *Ascaris lumbricoides*. Science. 1994; 265(5174): 954–956. 8052853 10.1126/science.8052853

[62] Pilotte N, Torres M, Tomaino FR, Laney SJ, Williams SA. A TaqMan-based multiplex real-time PCR assay for the simultaneous detection of *Wuchereria bancrofti* and *Brugia malayi*. Mol Biochem Parasitol. 2013; 189(1–2): 33–37. 10.1016/j.molbiopara.2013.05.001 23669148

[63] Tran AC, Halse TA, Escuyer VE, Musser KA. Detection of *Mycobacterium avium* complex DNA directly in clinical respiratory specimens: opportunities for improved turn-around time and cost savings. Diagn Microbiol Infect Dis. 2014; 79(1): 43–48. 10.1016/j.diagmicrobio.2014.01.019 24612561

[64] Easton AV, Oliveira RG, O’Connell EM, Kepha S, Mwandawiro CS, Njenja SM, et al Multi-parallel qPCR provides increased sensitivity and diagnostic breadth for gastrointestinal parasites of humans: field-based inferences on the impact of mass deworming. Parasit Vectors. 2016; 9(1): 38 10.1186/s13071-016-1314-y 26813411 PMC4729172

[65] Schmaedick MA, Koppel AL, Pilotte N, Torres M, Williams SA, Dobson SL, et al Molecular xenomonitoring using mosquitoes to map lymphatic filariasis after mass drug administration in American Samoa. PLoS Negl Trop Dis. 2014; 8(8): e3087 10.1371/journal.pntd.0003087 25122037 PMC4133231

[66] Rao RU, Nagodavithana KC, Samarasekera SD, Wijegunawardana AD, Premakumara WD, Perera SN, et al A comprehensive assessment of lymphatic filariasis in Sri Lanka six years after cessation of mass drug administration. PLoS Negl Trop Dis. 2014; 8(11): e3281 10.1371/journal.pntd.0003281 25393404 PMC4230885

[67] Lamberton PH, Cheke RA, Winskill P, Tirados I, Walker M, Osei-Atweneboana MY, et al Onchocerciasis transmission in Ghana: persistence under different control strategies and the roll of the simulidd vectors. PLoS Negl Trop Dis. 2015; 9(4): e0003688 10.1371/journal.pntd.0003688 25897492 PMC4405193

[68] Rodríguez-Pérez MA, Lilley BG, Domínguez-Vázquez A, Segura-Arenas R, Lizarazo-Ortega C, Mendoza-Herrera A, et al Polymerase chain reaction monitoring of transmission of *Onchocerca volvulus* of two endemic states in Mexico. Am J Trop Med Hyg. 2004; 70(1): 38–45. 14971696

[69] Rodríguez-Pérez MA, Gopal H, Adeleke MA, De Luna-Santillana EJ, Gurrola-Reyes JN, Guo X. Detection of *Onchocerca volvulus* in Latin American black flies for pool screening PCR using high-throughput automated DNA isolation for transmission surveillance. Parasitol Res. 2013; 112(11): 3925–3931. 10.1007/s00436-013-3583-0 24030195

[70] Fung MS, Xiao N, Wang S, Carlton EJ. Field evaluation of a PCR test for *Schistosoma japonicum* egg detection in low-prevalence regions of China. Am J Trop Med Hyg. 2012; 87(6): 1053–1058. 10.4269/ajtmh.2012.12-0177 23109374 PMC3516074

[71] Kure A, Mekonnen Z, Dana D, Bajiro M, Ayana M, Vercruysse J, et al Comparison of individual and pooled stool samples for the assessment of intensity of *Schistosoma mansoni* and soil-transmitted helminth infections using the Kato-Katz technique. Parasit Vectors. 2015; 8:489 26400064 10.1186/s13071-015-1101-1 PMC4581403

[72] Speich B, Knopp S, Mohammed KA, Khamis IS, Rinaldi L, Cringoli G, et al Comparative cost assessment of the Kato-Katz and FLOTAC techniques for soil-transmitted helminth diagnosis in epidemiological surveys. Parasit Vectors. 2010; 3:71 10.1186/1756-3305-3-71 20707931 PMC2936391

[73] Markoulatos P, Siafakas N, Moncany M. Multiplex Polymerase Chain Reaction: A Practical Approach. J Clin Lab Anal. 2002; 16(1): 47–51. 11835531 10.1002/jcla.2058 PMC6808141

